# Fourier spotting: a novel setup for single-color reflectometry

**DOI:** 10.1007/s00216-021-03802-w

**Published:** 2022-01-08

**Authors:** Johannes Siegel, Marcel Berner, Juergen H. Werner, Guenther Proll, Peter Fechner, Markus Schubert

**Affiliations:** 1 Institute for Photovoltaics, University of Stuttgart, Pfaffenwaldring 47, Stuttgart, 70569 Germany; 2 BioCopy GmbH, Elzstrasse 27, Emmendingen, 79312 Germany

**Keywords:** Single-color reflectometry, Fourier spotting, Light modulation, Biosensor

## Abstract

Single-color reflectrometry is a sensitive and robust detection method in optical biosensor applications, for example for bioanalysis. It is based on the interference of reflected monochromatic radiation and is label free. We present a novel setup for single-color reflectometry based on the patented technology of Berner et al. from 2016. Tilting areas of micro-mirrors allow us to encode the optical reflection signal of an analyte and reference channel into a particular carrier frequency with the amplitude being proportional to the local reflection. Therefore, a single photodiode is sufficient to collect the signals from both channels simultaneously. A 180^∘^ phase shift in the tilt frequency of two calibrated micro-mirror areas leads to a superposition of the analyte and reference signal which enables an efficient reduction of the baseline offset and potential baseline offset drift. A performance test reveals that we are able to detect changes of the refractive index *n* down to Δ*n* < 0.01 of saline solutions as regents. A further test validates the detection of heterogeneous binding interaction. This test compromises immobilized testosterone-bovine serum albumin on a three-dimensional layer of biopolymer as ligand and monoclonal anti-testosterone antibodies as analyte. Antibody/antigen binding induces a local growth of the biolayer and change in the refractive index, which is measured via the local change of the reflection. Reproducible measurements enable for the analysis of the binding kinetics by determining the affinity constant *K*_A_ = 1.59 × 10^− 7^ M^− 1^. In summary, this work shows that the concept of differential Fourier spotting as novel setup for single-color reflectometry is suitable for reliable bioanalysis.

**Supplementary Information:**

The online version contains supplementary material available at 10.1007/s00216-021-03802-w.

**Figure:**
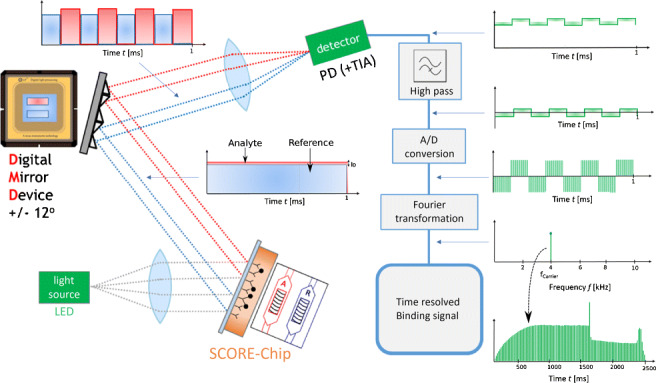
Graphical Abstract

## Introduction

Almost all biosensors are based on a two-component system to measure the binding affinity between the ligate (target molecule, e.g., antibody) and the ligand (inhibitor, e.g., antigen) [1]. Direct optical sensor systems detect this interaction without the need of labeling. Labeling is relatively expensive and time-consuming, and destroys the functionality of the target molecule [2]. Usually, in direct optical detection, heterogeneous test formats are used. Here, one partner (e.g., the antigen) is immobilized on a functionalized surface and the other (e.g., the antibody) is in liquid phase [3]. The functionalized surface consists of a transducer (e.g., glass) coated with thin layers with the recognition pattern on top. Reflectometry systems detect the reflected light, which is reflected at each interface of the layered system [4]. When the layer thicknesses are below the coherence length of the light source, one observes an interference pattern in the reflected light beams [5]. Due to the reaction of the antibody/antigen system, at least one of the layers on the transducer grows in the optical thickness and the reflection of the whole system changes.

Apart from static parameters of the light source (wavelength, angle of incidence, polarization), the interference pattern depends on the optical thickness *O* of the layers, with *O* = *n* × *d* which is given by the product of refractive index *n* and physical thickness *d* of the layer [2–5]. Binding interactions of the target molecules with the immobilized biolayer lead to an increase of *O* resulting in a change in the interference pattern. Conventional reflectometry interference spectroscopy (RIfS) uses a white light source and therefore depicts the reflected change of interference as a shift of the interference spectrum for all wavelengths [2, 3, 6]. Compared to other methods, RIfS has the advantage that, in principle, all kinds of transparent materials are appropriate as transducers. In contrast, other optical structures relying on evanescent field methods, e.g., surface plasmon resonance (SPR), need metallic layers that support surface plasmons like gold or grating couplers [2, 4, 7]. Also, in case of RIfS, in a first order, the temperature dependencies of optical thickness *n* and *d* cancel out [2–7]. These advantages make RIfS a fairly robust and easy-to-use biosensor [2]. Nevertheless, the evaluation of the reflection changes in RIfS for the complete spectrum makes the method tedious.

During the last years, a more elegant, new type of RIfS, single-color reflectometry (SCORE) (also known as 1-lambda-Reflectometry) was developed, which combines all advantages of RIfS with a much more simpler and portable measuring setup [7]. Instead of white light, monochromatic light is used for illuminating the binding interaction. Variations in the optical thickness *O* lead to a change of the wavelength’s reflected intensity, which is detected with a photodetector. In case of SCORE, a white light source, which often requires an additional waveguide, as well as the spectrometer, is not necessary. First investigations proved that the results from the monochromatic SCORE compare well to those obtained by multi-wavelength RIfS [7]; some studies achieve even better results [8]. Optimization of the wavelength of the monochromatic light source with respect to the biolayer thickness *d* enables a high detection sensitivity, leading to a maximum signal change for detecting binding interaction with SCORE. In contrast, in case of RIfS, the use of the whole multi-wavelength spectrum in the reflection measurements includes signals from wavelengths which barely change in reflection during the binding interaction of the antigen/antibody layers.

In principle, SCORE systems greatly facilitate practical applications and remove the need of costly spectrometers. Requiring only a single observation wavelength, SCORE can be combined with camera systems to observe thousands of individual analytic spots on one SCORE-Transducer in parallel. However, this technique requires costly low-noise scientific cameras, which produce a huge amount of data, during the measurement procedure. This data needs to be computed in order to extract the information of interest from the recorded picture series. The need for high computational power is contrary to the miniaturization towards low-cost portable SCORE platforms. The combination of SCORE with optical modulation methods from the field of single-pixel imaging seems to be a pathway to overcome the required high computational power due to conventional camera systems by reducing the recorded data to a single time signal. In general, single-pixel imaging describes the detection of an object with various modulation schemes so that a one-element detector is sufficient to recover the image [9]. These modulation schemes also allow encoding the optical signals of parallelly measured analytic SCORE spots to a single time signal. In their patent application, Berner et al. [10] described a novel differential modulation scheme aiming to enhance differential SCORE measurements that compares the optical signals of analytic spots against the signals of reference spots. Thereby, the reflected signal of each particular analytic spot is optically modulated with a unique carrier frequency. Additionally, the reflected signal of a reference spot is modulated with the same carrier frequency, but with a phase shift of 180^∘^. When the reflected signals of an analytic spot and its reference spot are unequal, this will cause a differential signal oscillating at the carrier frequency corresponding to this spot pair. The superposition of all signals is measured by a single-element detector and the differential signals can be recovered from the recorded sum signal using a Fourier transformation. Thus, this method is referred to as differential Fourier spotting (DFS).

This work presents a novel setup that implements the DFS method for SCORE measurements following the concept of Berner et al. [10] and a proof of concept for the newly developed DFS-SCORE by demonstrating measurements using a single pair of analytic and reference spots. Our performance test of DFS-SCORE uses saline solutions with various NaCl concentrations *c*, in order to test the sensitivity for local changes in refractive index *n*: With a limit of detection (LOD) of *LOD*_c_ = 0.5*%* and a limit of quantification (LOQ) of *LOQ*_c_ = 1.6*%*, the system is able to detect changes in refractive index *n* down to Δ*n* < 0.01. To demonstrate the system’s suitability as a biosensor, measurements of biological binding interactions are carried out. For this reason, testosterone-bovine serum albumin (BSA) is immobilized on a three-dimensional layer of biopolymer. In order to measure the binding interaction of anti-testosterone antibodies (monoclonal mice), which are contained in a liquid, this solution is injected over the three-dimensional layer. Stock solutions of antibodies *C*_A_ diluted in phosphate-buffered saline (PBS) of less than 300 μL are necessary as ligate volume. Reproducible measurements of time-resolved biological binding interaction demonstrate the ability of DFS-SCORE to be suitable as a biosensor to investigate molecular binding interactions.

## Experimental setup

### Reflectometric interference methods RIfS and SCORE

**Fig. 1:**
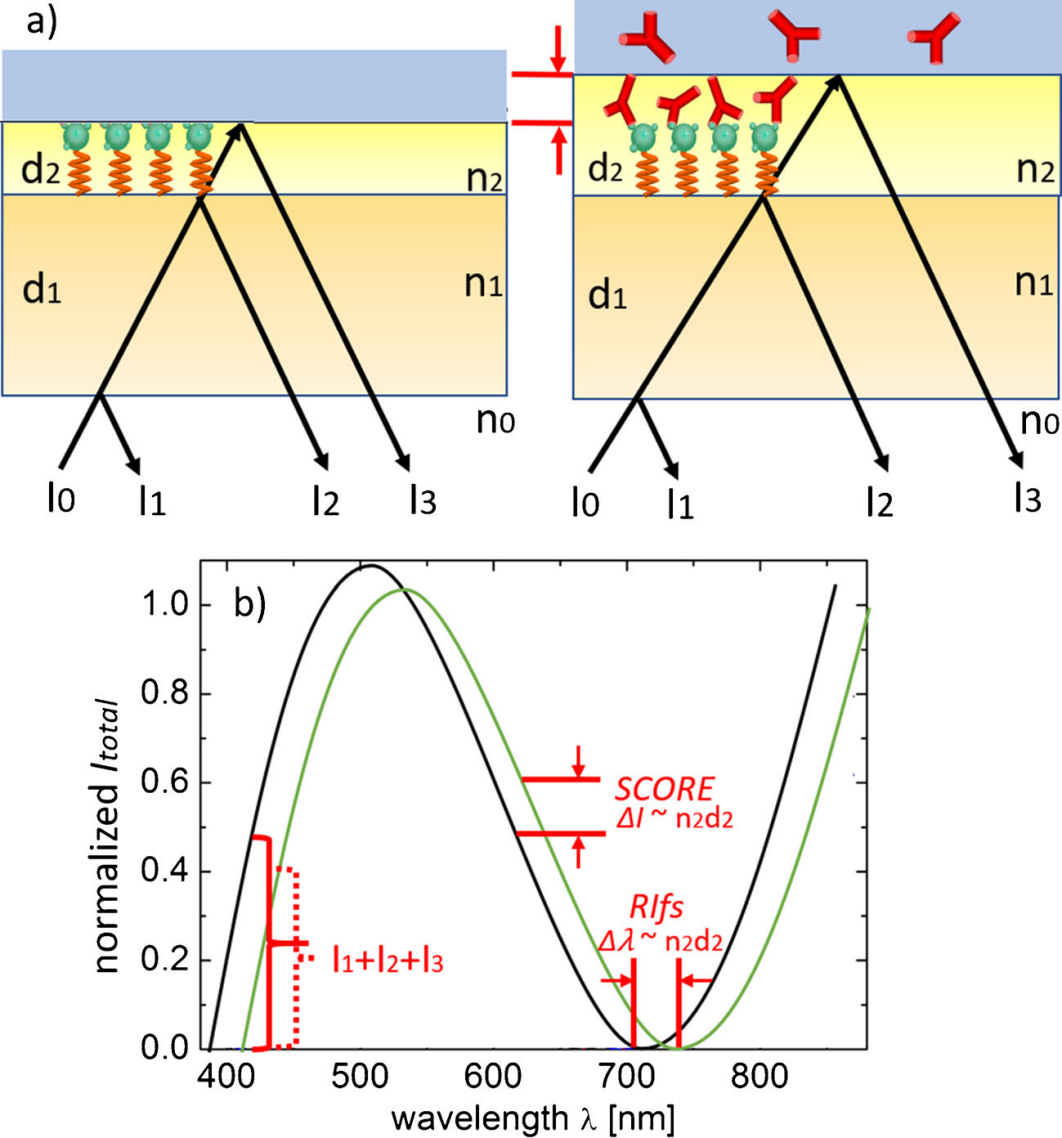
**a** Glass transducer chip with thickness *d*_1_ coated with recognition pattern of biopolymer *d*_2_ is illuminated from backside producing an interference pattern of the reflected light. The reflected signal changes due to the growth of the biolayer’s thickness *d*_2_ and refractive index with the antibodies from aqueous solution. **b** Growth of the biolayer changes the reflection around *I*_1_ + *I*_2_ + *I*_3_. In case of RIfS, reflection changes/shifts over the whole wavelength regime are observed. In case of the monochromatic SCORE, one evaluates the reflection change just for one single wavelength

Figure 1a and b demonstrate the setup of the region of interest, containing the transducer and the biolayer with the immobilized antigens. Figure 1a, which is not drawn to scale, shows the transducer chip with the glass of thickness *d*_1_ = 1 mm and the biopolymer *d*_2_ on top (left). During time *t*, the thickness is increased in the nanometer range as a consequence of the antibody/antigen binding (right) when the antibodies flow in an aqueous solution over the biolayer [8].

The light beam comes from the back side of the glass and undergoes partial reflection and transmission at each particular interface. The layer thickness *d*_2_ is below the coherence length of the LED light. Therefore, its thickness change Δ*d*_2_ leads to a stable, static interference pattern. The totally observed reflected signal has an intensity

1 $$ I = I_{1}+I_{2}+I_{3}+2 \sqrt{I_{2} I_{3}}  cos \left( \frac{4\pi O_{2}}{\lambda}\right).  $$

Here, *I*_1_, *I*_2_, and *I*_3_ are the partial light intensities from the glass, original biolayer, and biolayer with bonded molecules respectively [6]. The quantity *O*_2_ stands for the biolayer’s optical thickness and *λ* is the wavelength of the light source. Depending on the phase of the cosine wave in Eq. (1), the change of *O*_2_ during the binding interaction leads to an increase or decrease in *I* [8]. In our case, we use perpendicular incidence of the light beam.

Figure 1b shows the normalized (1) and compares the signal of RIfS and SCORE. The RIfS technique evaluates the optical thickness change Δ*O*_2_ with the wavelength shift Δ*λ* of the whole interference pattern [2, 3, 6, 7]. In contrast, SCORE measures Δ*O*_2_ from the reflection change Δ*I* at a single wavelength [7, 8]. The highest reflection change Δ*I* in SCORE is obtained if one uses the wavelength *λ* at the inflection point (around 620 nm in Fig. 1b of the cosine function). In contrast, the signal change is smallest at the maxima or the minima of

the spectrum. Thus, by choosing the optimal wavelength for a given original physical thickness *d*_2_ of the biopolymer gives SCORE a higher contrast when compared to RIfS [8].

### Setup and signal path of differential Fourier spotting

**Fig. 2:**
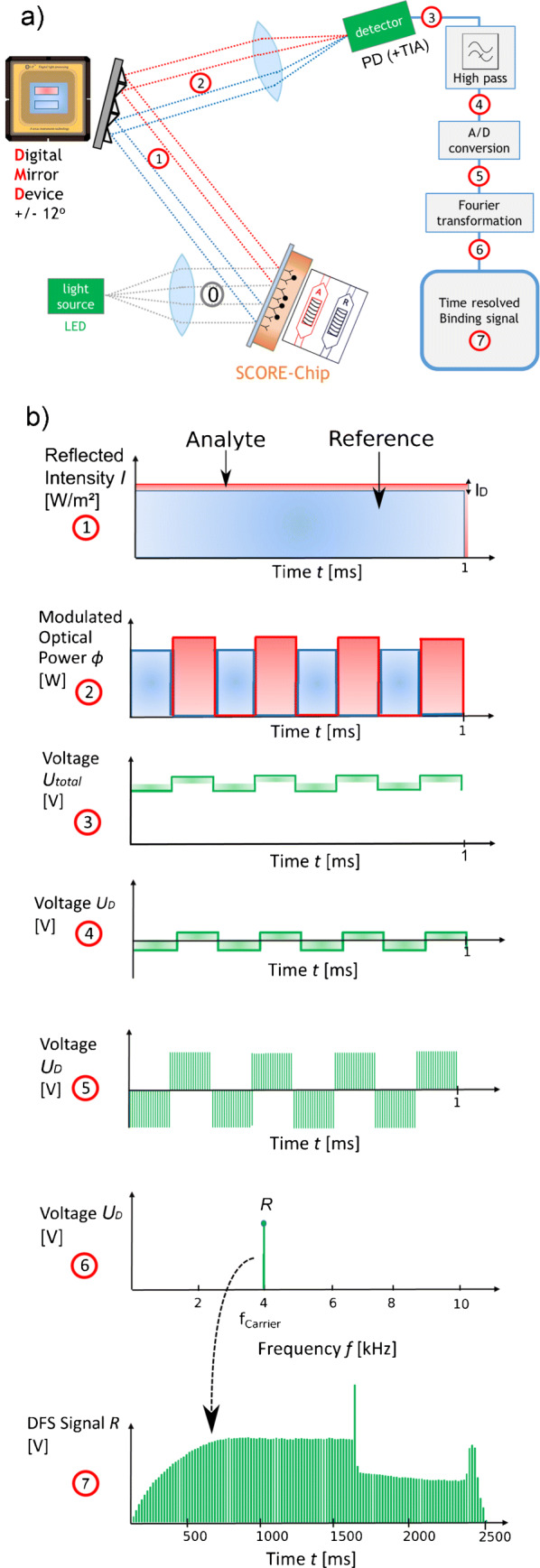
**a** Setup of DFS-SCORE. **b** Signal path of DFS-SCORE. Description in “Setup and signal path of differential Fourier spotting”

The basic structure of our differential Fourier spotting (DFS) system to run SCORE measurements is divided into three parts: illumination, modulation, and processing. For a traceable signal path of the DFS, the setup itself combined with the signal path is depicted in Fig. 2a and b. Figure 2a shows the schematic overview of the experimental setup, whereas Fig. 2b shows the signal path. Both figures are connected over the circled numbers showing the positions of the current signal which are also used for the following explanation as numbers , , , etc.

Illumination: The LED as a quasi-monochromatic light source illuminates a glass transducer chip coated with a biopolymer layer including immobilized antigens (ligand) on top. The chip is placed on a flow cell, which consists of two separate channels: analyte (A) and reference (R) channels with running antibody solution (ligate) and buffer solution, respectively. Number  contains both reflected signals, which are indicated as *I*_A_ (red line) and *I*_R_ (blue line) in both Fig. 2a and b. Regarding (1), the small difference signal *I*_D_ is expressed as

2 $$ I_{\mathrm{D}} = I_{\mathrm{A}} - I_{\mathrm{R}} =  I_{\text{int}} \left[ cos \left( \frac{4\pi O_{\mathrm{A}}}{\lambda} \right)- cos \left( \frac{4\pi O_{\mathrm{R}}}{\lambda}\right) \right]  $$

with $I_{\text {int}}= 2 \sqrt {I_{2} I_{3}} \sim I_{0}$ and stems from the growth in optical thickness as a consequence of the ligate/ligand binding and, thus, is the signal of interest.

Modulation: The reflected intensities *I*_A_ and *I*_R_ are focussed on a digital mirror device (DMD), which contains a surface of micro-mirrors. Two surface areas *S*_A_ and *S*_R_ of micro-mirrors on the DMD modulate *I*_A_ as well as *I*_R_ with the same tilting frequency, also denoted as carrier frequency. Number  shows the resulting modulated optical power signals Φ_A_ = *I*_A_*S*_A_ and Φ_R_ = *I*_R_*S*_R_, both modulated with the same carrier frequency of 4 kHz but phase-shifted with 180^∘^ in order to later subtract *I*_R_ from *I*_A_. The superposition of these two modulated optical signals falls onto one photodiode (PD) and generates a photocurrent.

Processing: On the electronic side, the superimposed photocurrent is converted into an electrical voltage *U*_Total_  with the help of a *T* rans*i* mpedance *A* mplifier (TIA). The voltage signal *U*_total_ consists of a DC and an AC component, where the AC component is proportional to the desired signal of interest *I*_D_. The DC component is equal to the signal offset and can be suppressed by a high-pass filter. As a consequence, solely the AC component of *U*_total_, corresponding to *U*_D_, remains as represented in number  . The voltage *U*_D_ is then digitized via an analog-to-digital converter (number ). The now discrete-time signal is transferred to the frequency domain by Fourier transformation (number ). Here, the amplitude of *U*_D_ is obtained at the carrier frequency and extracted throughout the total measurement process forming the time-dependent DFS signal *R* in number .

### Optical setup

Our actual optical setup exhibits most features of the schematic setup of Fig. 2a. In contrast to Fig. 2a, the optical paths for illumination  and reflected signals  are perpendicular to the analyte spots coupled via a beam splitter. With the use of a telecentric lens, the light beams of the LED light source (MCEP-070 Series) are formed as parallel light beams, which is required for SCORE at the point of interest.

**Fig. 3:**
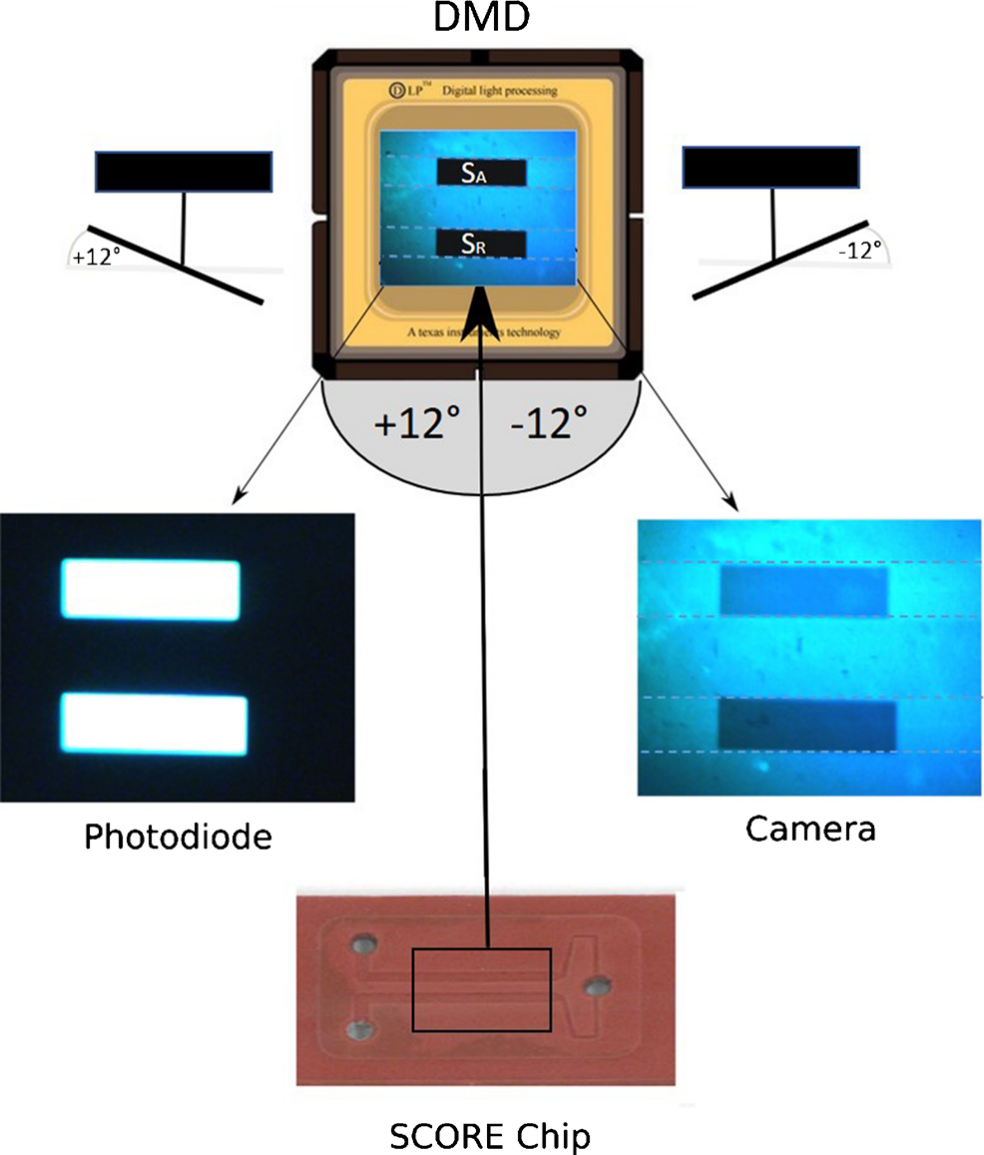
Scheme of the image distribution at DFS-SCORE. The reflected light from the SCORE Chip is imaged on the whole area of the DMD. There, the micro-mirrors of area *S*_A_ and *S*_R_ guide the light in either 12^∘^ or − 12^∘^ direction, where the rest of the image is in constant − 12^∘^ direction. The photodiode detects the reflected light of *S*_A_ and *S*_R_ in 12^∘^ direction where the light of *S*_A_ and *S*_R_ in − 12^∘^ direction plus the rest of the image is detected by the camera. This optical structure allows the tracking of the calibration and measuring process with two detection units without optical separation and loss of light intensity

Figure 3 shows the reflected light which is imaged on the DMD (DLP7000, Texas Instruments) with a ratio of 4:1. The mirror areas *S*_A_ and *S*_R_ on the DMD reflect and modulate the light signals *I*_A_ and *I*_R_ with their phase-shifted tilt frequency to a silicon PD (PDA 100A2, Thorlabs), which is placed at an angle of + 12^∘^ to the DMD, and to a CMOS camera (DFK 42BUc03, Imagingsource), which is placed at an angle of − 12^∘^ to the DMD. The rest of the flow cell image is guided constantly in − 12^∘^ direction to the camera. While the PD is the measuring unit, the camera line enables a live optical view of the whole flow cell including both channels. This optical setup allows the tracking of the measuring process with two detection units without optical separation and loss of light intensity. The whole optical structure of DFS-SCORE is designed using *ZEMAX* software for maximum throughput from the SCORE Chip to the PD. The setup is completely covered with black hardboard slides to avoid measurement errors caused by ambient light.

### Mirror calibration

The LED shows a spatial intensity distribution, which leads to an imbalance of the optical power signals Φ_A_ and Φ_R_. This imbalance can be resolved with adjusting the size of the surface areas *S*_A_ and *S*_R_ in terms of their amount of micro-mirrors [10], so that

3 $$ \Upphi_{\text{int}}=S_{A} I_{\mathrm{A}} = S_{R} I_{\mathrm{R}}  $$

**Fig. 4:**
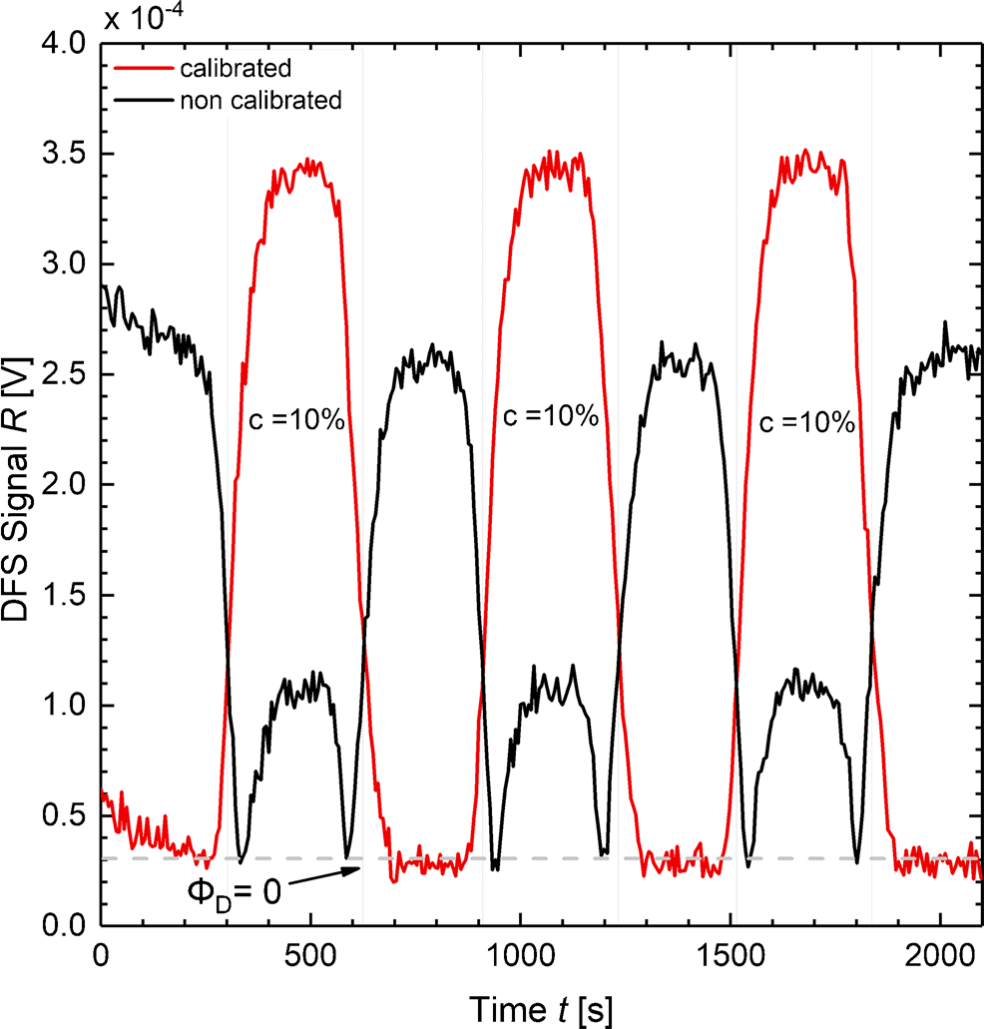
Measurement of a sequence of three identical sample concentrations *c* alternating with buffer is modulated with non-calibrated and calibrated offset of Φ_D_. For the non-calibrated measurement (black plot), *R* inverts at Φ_D_ = 0. In comparison, the offset calibrated measurement (red plot) is free of this signal inversion

Thus, for equal optical thicknesses *O*_*A*_ = *O*_*R*_ at both observed spots, the differential optical power

4 $$ \Upphi_{\mathrm{D}}=\lvert \Upphi_{\mathrm{A}}-\Upphi_{\mathrm{R}} \rvert=\Upphi_{\text{int}} \displaystyle\left\lvert cos \left( \frac{4 \pi O_{\mathrm{A}}}{\lambda} \right)- cos \left( \frac{4 \pi O_{\mathrm{R}}}{\lambda} \right) \right\rvert  $$

is calibrated to be Φ_D_ = 0. As the current setup can only measure the absolute amplitude of Φ_D_ but no phase information, this offset calibration is mandatory to prevent signal inversion, due to a change of sign of Φ_A_ −Φ_R_. Figure 4 depicts measured DFS signals *R* with and without a calibrated offset of Φ_D_ for a sequence of three identical sample concentrations *c* alternating with buffer. For the non-calibrated measurement, a decrease of *O*_A_ leads to a decrease of *R* until *R* = 0.3 × 10^− 4^ V is reached at approximately *t* = 320 s. Then, any further decrease of *O*_A_ leads to an increase of *R*, due to signal inversion. In comparison, the offset calibrated measurement shown in Fig. 4 is free from this signal inversion. More details regarding the calibration procedure are given in Supplementary information.

### Microfluidic system

The microfluidic system is divided into two lines: the analytic line A with the analyte to detect and the reference line R with the buffer solution. The analytic line with a 6-way valve system enables the measurement of five samples. Liquid handling is achieved by a peristaltic micro-pump (ISM832C, ISMATEC), which generates the volume flow in both lines. For each of the analytic line as well as of the reference line, a bubble trap is installed (LVF-KBT-S, DARWIN). The region of interest (SCORE-Chip) is about 5 × 5 mm^2^ and composed of 1-mm glass holding the fluidic cell of polydimethylsiloxane (PDMS) including two separate micro-channels (length: 10^3^μm, width: 10^2^μm, height: 10μm) with two entries for both lines and one common exit. A spring clamp clings the sandwich of PDMS and glass with a proper pressure preventing distortion of the sample spot and ensuring constant channel heights for homogeneous flow rates. An illustration of the fluidic system is provided in Fig. 9 in Supplementary information.

## Binding interaction

Biomolecular binding interactions base on the specific affinity between the binding partners ligand and ligate. Our DFS-SCORE is a direct optical system, which uses a heterogeneous test format: the ligand is immobilized and the ligate is in liquid phase. Binding interaction between two partners is controlled by two effects: mass transport and binding kinetics. First, the mass transport of the ligate is caused by the diffusion of the ligate to the surface which can be described by the first Fick’s law [1]. Secondly, at the surface, the binding kinetics is described by the exponentially time-dependent association and dissociation of the ligate with its immobilized ligand [1]. For a more detailed description of the mass transport and binding kinetics, see Supplementary information.

## Materials

### Transducer

Proprietary standard test transducers were provided by BioCopy GmbH, Emmendingen (Germany). Briefly, they consist of an optically modified glass slide of 1-mm substrate thickness covalently modified with polyethylene glycol (PEG) as shielding against non-specific binding. This polymer was activated using active ester chemistry for a covalently immobilization of testosterone-BSA.

### Analytes

Biomolecular binding interaction is conducted with monoclonal anti-testosterone antibodies as recognition ligate. Extracted from immune cells of a mouse’s body, monoclonal antibodies have high specificity and affinity and can be produced in large quantities which makes them convenient for laboratory investigations. Stock solution of monoclonal anti-testosterone antibodies is pipetted in phosphate-buffered saline (PBS) to produce ligate samples of various concentrations. The stock solution consists of 50 μg antibodies diluted in 50 μL PBS and is stored in aliquots of 50 μL at − 20^∘^C. Mixed solutions are stored for max. 14 days at 5^∘^C. To detach the antibodies from the ligand sites, a regeneration solution of 6M guanidine hydrochloride solution (GuHCl, pH 1.5) is applied which has a strong denaturing effect on proteins. GuHCl as a chaotropic salt might cause denaturation of the BSA itself. However, this should have a minor influence on the affinity of the antibody/antigen interaction since the covalent bond between BSA and testosterone should not be affected by this regeneration. Furthermore, the BSA-testosterone is covalently immobilized to the transducer surface and will therefore not suffer from regeneration process as well.

### Measuring procedure

Here, we report on two sets of measure protocols. As a first set, saline solutions of various NaCl concentrations are used for evaluating the performance of DFS-SCORE. Equation (1) shows that SCORE always detects changes of the effective biolayer thickness *n*_2_*d*_2_. Therefore, saline solutions with varying concentrations are well suited for simulating changes in *n*_2_ which might similarly originate from the change of refractive index of biopolymer in case of attaching analyte molecules to the immobilized target. Due to a lack of binding interaction when using saline solution, the physical thickness *d*_2_ is constant.

The second set of experiments characterizes biological interactions between testosterone antibodies (monoclonal mice) and testosterone-BSA antigens. Each transducer chip is first cleaned with GuHCl applied for 5 min. Then, PBS is applied to set the base level following the ligate to bind with the immobilized ligand. All measurements are performed at room temperature with a flow rate of 20 μL/min.

## Results

### Performance test

**Fig. 5:**
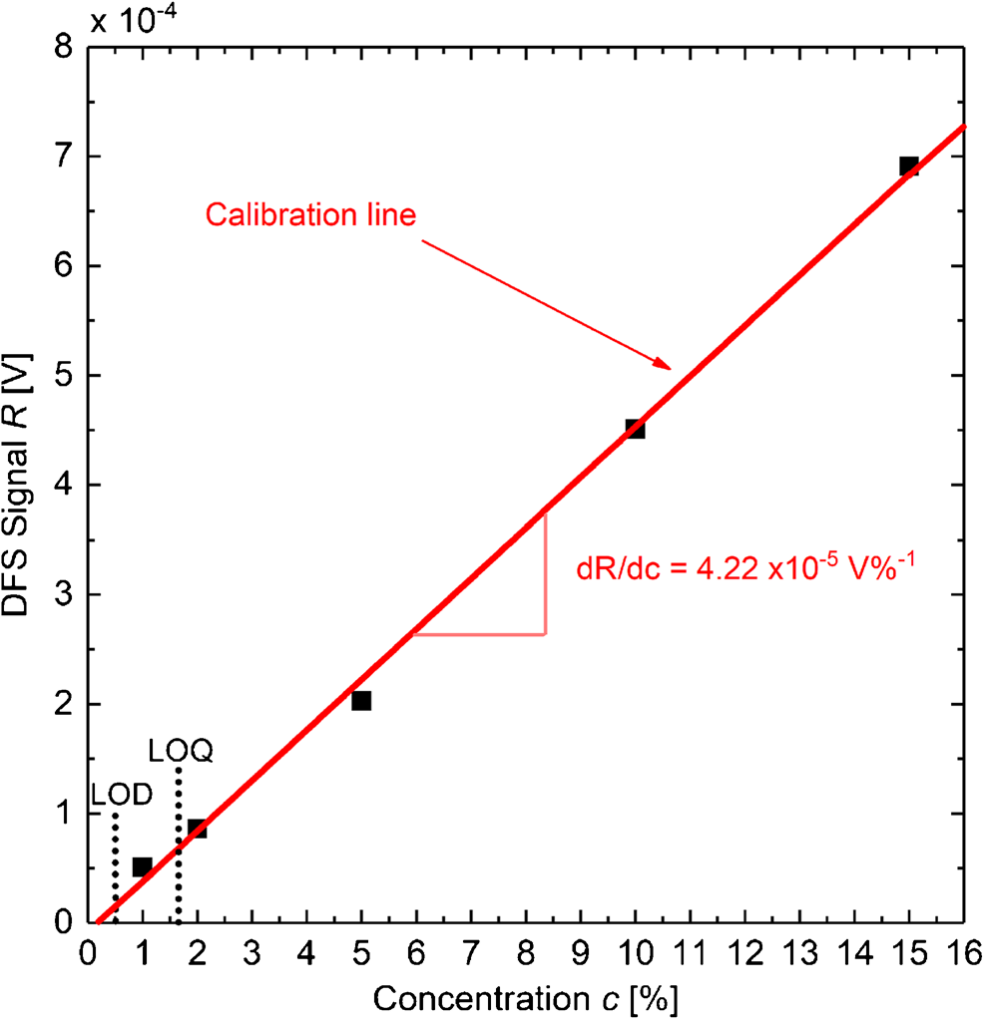
DFS Signal *R*(*t*) for different NaCl concentrations *c* = 15*%*, 10 *%*, 5*%*, 2*%*, and 1*%* in the saline solution. The linear calibration indicates a slope of 4.22 × 10^− 5^*V%*^− 1^ and a *LOD*_c_ = 0.5*%* and *LOQ*_c_ = 1.6*%*

A reliable biosensor needs high sensitivity, stability, and reproducibility. The key challenge within this work is to bring the novel detection scheme for SCORE from theory into practical operations for observing biological binding events. Prior to the detection of binding events, a performance test with saline solution is therefore undertaken to the constraints of the sensor. For that purpose, the limit of detection (LOD) and the limit of quantification (LOQ) are utilized. According to the International Conference on Harmonisation (ICH), the LOD is the lowest amount of analyte in a sample, which can be detected, whereas the LOQ describes the lowest amount of analyte in a sample, which can be quantitatively determined with suitable precision and accuracy [11]. A common method to quantify both limits is to calculate

5 $$ LOD=3.3 \times \frac{\sigma_{\text{bas}} }{dR/dc}  $$

6 $$ LOQ= 10 \times \frac{\sigma_{\text{bas}} }{dR/dc}  $$

with *σ*_bas_ as standard deviation of the baseline and *dR*/*dc* as slope of the calibration curve [11]. Figure 5 shows the DFS signal *R* for different saline solution concentrations *c* of 15*%*, 10*%*, 5*%*, 2*%*, and 1*%*, from Fig. 10 in Supplementary information. A linear calibration line fits well, yielding a slope of *dR*/*dc* = 4.22 × 10^− 5^ V*%*^− 1^. According to Eqs. 5 and 6, these values with *σ*_bas_ determined in Fig. 10 lead to *LOD*_*c*_ = 0.5*%* and *LOQ*_*c*_ = 1.6*%* corresponding to a sensitivity Δ*n* < 0.01 in fluids [12].

### Binding interaction test

**Fig. 6:**
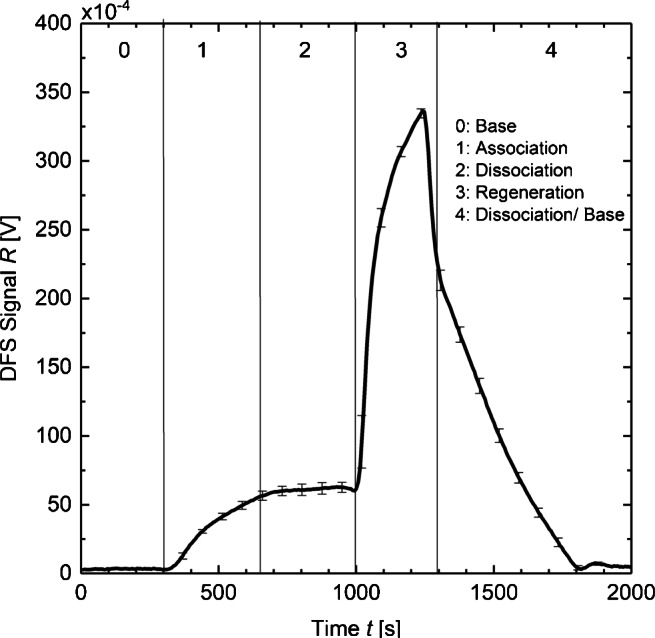
Complete sensorgram of a monoclonal anti-testosterone antibodies (*C*_A_ = 133 nM) with BSA-antigen. After setting the baseline level by PBS (0), association occurs by antibodies-solution (1). Dissociation (2) and detaching of the antibodies (3) lead to a regeneration of the transducer chip where PBS sets the baseline level again (4)

In the next step, Fig. 6 proves the suitability of DFS-SCORE to investigate biological binding analyses. For this purpose, we execute measurements of time-resolved binding interactions between antibody ligates and antigen ligands. Various concentrated monoclonal anti-testosterone antibody solutions are flushed over a fixed concentration of testosterone-BSA antigens, which are immobilized on a glass transducer. The binding interaction in Fig. 6 uses an example antibody concentration *C*_A_ = 133 nM and has the following numbered phases:

0. Base phase: Buffer solution (PBS) sets the baseline.
1. Association phase: Antibodies bind to antigens on the chip transducer which leads to an increase in optical thickness.
2. Dissociation phase: PBS is applied to dissociate the antibodies ligates from the antigen ligands. Due to the low dissociation of the testosterone monoclonal antibodies, a decrease in the DFS signal is not observable.
3. Regeneration phase: Regeneration solution (GuHCl) denatures the binding sites and hence releases the antibodies.
4. PBS washes the antibodies away and sets the baseline level again.

All solutions are pumped in a time period of 5 min with a ligate volume below 300 μL.

**Fig. 7:**
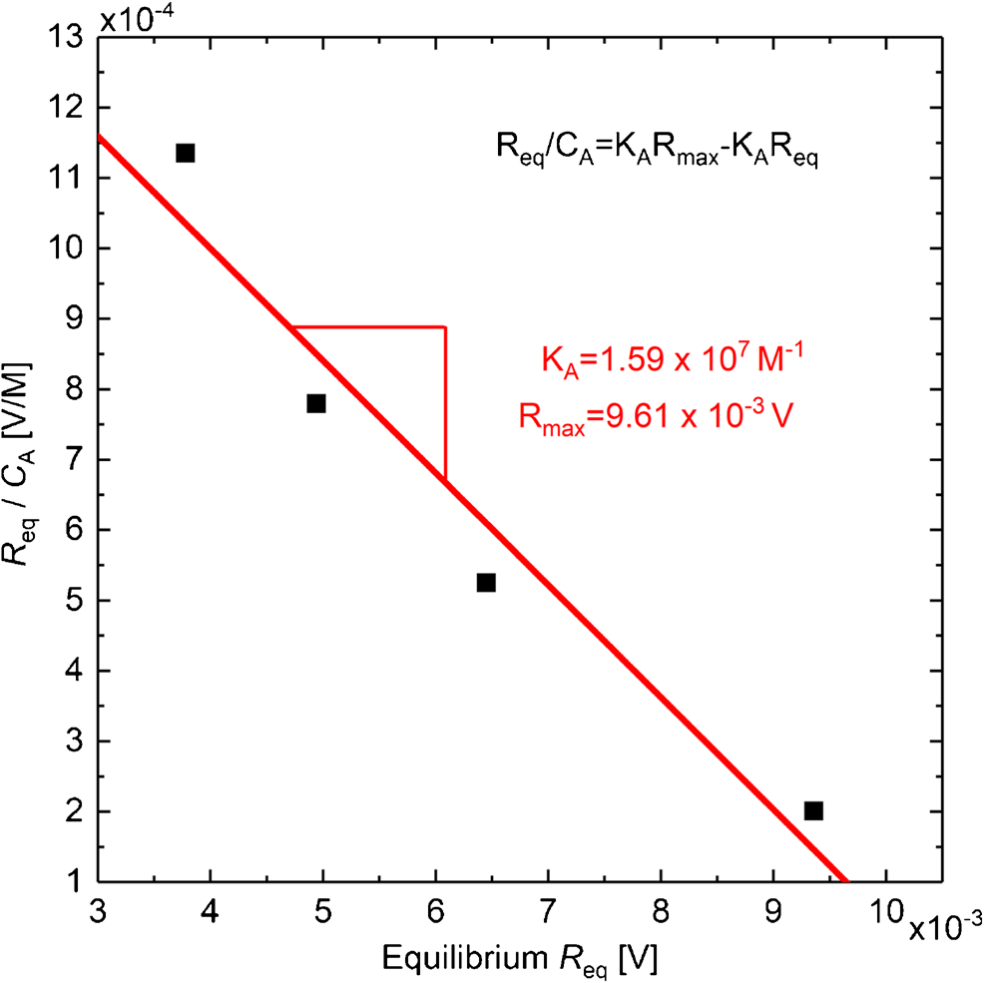
Scatchard plot to determine *R*_A_ and $R_{\max \limits }$ with help of a regression line. From the slope one can obtain *K*_A_ = 1.59 × 10^7^ M^− 1^ and $R_{\max \limits }=9.61 \times 10^{-3}$ V from the abscissa

As already noticed in Fig. 6, the theoretical decrease of the DFS signal in the dissociation phase does not occur. However, to determine the binding kinetics of the testosterone system nevertheless, the case of equilibrium binding state can be utilized. Therefore, we measured the binding curves of antibody concentrations *C*_A_ = 33 nM, 66 nM, 133 nM, and 466 nM and determined the reflection signals at equilibrium *R*_eq_ (see Figs.11 and 12 in Supplementary information). Figure 7 shows the ratio *R*_eq_/*C*_A_ plotted against *R*_eq_ using the Scatchard equation

7 $$ \frac{R_{\text{eq}}}{C_{\mathrm{A}}}=K_{\mathrm{A}} R_{\max}-K_{\mathrm{A}}R_{\text{eq}}  $$

which is a very common linear transformation of binding data [13]. One obtains the association constant *K*_A_ = 1.59 × 10^7^ M^− 1^ from the slope of a regression line and the $R_{\max \limits }=9.61 \times 10^{-3}$ V from the abscissa. For comparison purposes, the value of *K*_A_ should be interpreted with caution due to the general difficulties of evaluating binding kinetics. Possible deviations from the exponential kinetik behavior can occur by multivalent ligates *C*_A_ which deviates from the 1:1 stoichiometry assumption (see Eqs. 8 and 14 in Supplementary information) and/or spatial heterogeneity of the ligand sites to be approached by ligate which becomes significant as ligand sites approach saturation [14]. Furthermore, a study of 2019 found out that different measurement systems can deviate up to a factor of 20 for the same analyte [15]. Considering this, variations in determining *K*_A_ with different setups are more than expectable. Nevertheless, the reproducibility of binding interaction measurements shows the potential of DFS-SCORE to analyze further biomolecular binding systems.

## Conclusion/outlook

This work has given a proof for the suitability of DFS-SCORE as a novel and reliable setup of SCORE measurements. First, we analyzed the performance of the system in terms of optical resolution with saline solution as test sample due to their simplicity as recognition element. The system detects changes of the refractive index *n* down to Δ*n* < 0.01. Furthermore, the differential modulation of calibrated micro-mirror areas suppresses the baseline offset and potential baseline offset drift to a minimum. The DFS-SCORE method allows one to build automated biosensors demonstrated by detecting heterogeneous binding interactions of various monoclonal anti-testosterone antibody solutions (< 300 μL) with immobilized testosterone-BSA antigens. The analysis of the binding kinetic with determining *K*_A_ = 1.59 × 10^7^ M^− 1^ shows the ability of DFS-SCORE to investigate biological binding systems. However, the fully potential of DFS-SCORE is not tapped yet. An algorithm to fully automate the mirror calibration would result in a more reliable and time-saving mirror calibration. In addition to the mirror calibration, a phase synchronization of the modulated optical power Φ_D_ and the sampled voltage *U*_D_ could also correct the possible signal inversion for an imbalance of Φ_A_ and Φ_R_. Furthermore, spatial resolution can be achieved by modulating multiple sample spots with multiple carrier frequencies simultaneously. This would allow high-throughput measurements with a single photodiode as detector in a quick and cost-effective way for possible applications in diagnostics. Therefore, the limitation factors of the DMD in form of surface area and carrier frequency range as well as the influence of the carrier frequencies itself are worth to be investigated further.

## Electronic supplementary material

Below is the link to the electronic supplementary material.

(PDF 1.09 MB)

## Data Availability

The datasets generated during and/or analyzed during the current study are available from the corresponding author on reasonable request.

## Declarations

### Source of biological material

Testosterone (3-CMO-BSA) Mouse Monoclonal Antibody (BM2076) were obtained from Origene, 32052 Herford, Germany.
