# Supplementary material for: Fourier spotting: a novel setup for single-color reflectometry
Source: Anal Bioanal Chem. 2022 Jan 8;414(5):1787–96. doi: 10.1007/s00216-021-03802-w (PMC8791914; doi:10.1007/s00216-021-03802-w)
Supplement: Supplementary file 1 — (PDF 1.09 MB) [file 216_2021_3802_MOESM1_ESM.pdf]

Supplementary Information

Fourier Spotting: A Novel Setup for Single Colour Reflectometry

Johannes Siegel[1*], Marcel Berner[1], Juergen H. Werner[1], Guenther Proll[2], Peter Fechner[2] and Markus Schubert[1]

[1*]Institute for Photovoltaics, University of Stuttgart, Pfaffenwaldring 47, Stuttgart, 70569, Germany.
[2]BioCopy GmbH, Elzstrasse 27, Emmendingen, 79312, Germany.

Corresponding author(s). E-mail(s): johannes.siegel@ipv.uni-stuttgart.de;
Contributing authors: mb@innovativepyrotechnik.de; juergen.werner@ipv.uni-stuttgart.de; guenther.proll@biocopy.de; peter.fechner@biocopy.de; Markus.Schubert@ipv.uni-stuttgart.de;

Mirror Calibration

A mirror calibration is undertaken prior every measurement to align the modulated optical power signals $\Phi_A = I_A S_A$ and $\Phi_R = I_R S_R$ in order to obtain $\Phi_D = |\Phi_A - \Phi_R| = 0$ and hence to reduce baseline level to a minimum. Figure S1 (not to scale) depicts the process of a mirror calibration. In this case, the surface areas S_A and S_R consist of 400×44 mirrors with a single mirror area of $93\mu m^2$ leading to a surface of 1.6 mm^2 for each area. Single mirrors blocks of M=0.004 mm^2 for both mirror areas are added or removed until $\Phi_D = 0$ is reached at $R = 10^{-4}$ V with $\Delta S_A = -3$ M,$\Delta S_R = 0$ M corresponding to a maximum suppression of the baseline offset. It has to be added, that the magnitude of $\Phi_D = 0$ can slightly vary depending on the original sizes of S_A and S_R.

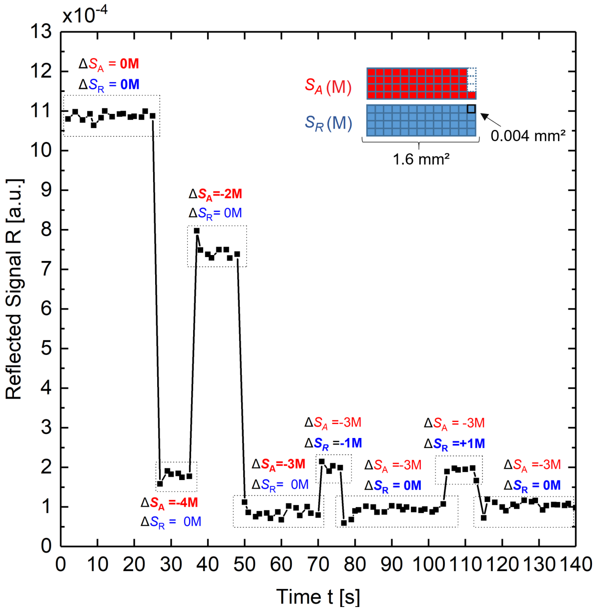

Fig. S1 Time resolved mirror calibration to adjust the micro mirror surfaces S_A and S_R during the baseline phase of both channels. Single mirrors blocks M for S_A and S_R are added/removed manually until the maximum suppression of the baseline offset is obtained at $R = 10^{-4}$ with $\Delta S_A = -3$M,$\Delta S_R = 0$M.

Binding kinetics

The law of mass action for equilibrium reaction of the binding at the surface may be described with

$$C_{\mathrm{A}} + C_{\mathrm{B}} \underset{\mathrm{k_d}}{\overset{\mathrm{k_a}}{\rightleftarrows}} C_{\mathrm{AB}}, \tag{8}$$

where C_{A} is the concentration of the free ligate mol/l = M, C_{B} the concentration of the free ligand (on the surface) [M], C_{AB} the concentration of the ligate-ligand-complex [M], and k_{a}, k_{d} are the respective association [s/M] and dissociation [1/s] rate constants [14]. In our case, the ligate solution is continual replenished, therefore C_{A} remains constant. Therefore, equation (8) can be simplified to a pseudo first order reaction. The time dependence for the formation of the complex is thus expressed as

$$\frac{dC_{\mathrm{AB}}}{dt} = k_{\mathrm{a}} C_{\mathrm{A}} C_{\mathrm{B}} - k_{\mathrm{d}} C_{\mathrm{AB}} \tag{9}$$

with the assumption that the ligate maintains its initial value. The first part of equation (9) describes the formation, while the second part describes the decay of the complex C_{AB}. Since C_{AB} is proportional to the optical thickness of the biolayer and therefore proportional to the reflected light intensity $R(t)$, equation (9) may be rewritten as

$$\frac{dR(t)}{dt} = k_{\mathrm{a}} C_{\mathrm{A}} (R_{\mathrm{max}} - R(t)) - k_{\mathrm{d}} R(t), \tag{10}$$

where $R(t)$ denotes the reflection signal at the time t and R_{max} the maximal reflection signal in case of all available ligand sites were saturated (i.e. the reflection signal corresponding to $C_{\mathrm{B}} = 0$ M). The solution of this differential equation for the reflection signal is

$$R(t) = \frac{k_{\mathrm{a}} C_{\mathrm{A}} (R_{\mathrm{max}} (1 - \exp^{-k_{\mathrm{s}} t}))}{k_{\mathrm{s}}}, \tag{11}$$

where

$$k_{\mathrm{s}} = k_{\mathrm{a}} C_{\mathrm{A}} + k_{\mathrm{d}} \tag{12}$$

expresses the observable pseudo-first-order rate constant k_{s}. For very long times t, equation (11) leads to

$$R(t \to \infty) = R_{\mathrm{eq}} = \frac{k_{\mathrm{a}} C_{\mathrm{A}} R_{\mathrm{max}}}{k_{\mathrm{s}}}, \tag{13}$$

with R_{eq} as reflection signal at equilibrium. With this new variable, equation (11) can be rewritten as

$$R(t) = R_{\mathrm{eq}} (1 - \exp^{-k_{\mathrm{s}} t}), \tag{14}$$

describing the binding interaction as Langmuir adsorption [14]. In chemistry, the affinity constant K_{A} describes a specific constant in which a molecule binds to its receptor and can be described as equilibrium constant solved by the mass law from equation (8)

$$K_{\mathrm{A}} = \frac{k_{\mathrm{a}}}{k_{\mathrm{d}}} = \frac{C_{\mathrm{AB}}}{C_{\mathrm{A}} C_{\mathrm{B}}}, \tag{15}$$

with K_{A} as affinity constant [M^{-1}] [1].

Mass transport

In our investigation of heterogeneous binding interaction, the ligate molecules in the solution first have to diffuse from the bulk solution to the surface with the immobilized ligand attached. The diffusion is driven by concentration gradient of C_{A} between the bulk and the surface. As a consequence, at the beginning of the experiment, when the concentration gradient still exists, the binding interaction is dictated by mass transport in the liquid. Therefore, the reflection deviates from the simple exponential behavior predicted by equation (14).

Supplementary figures

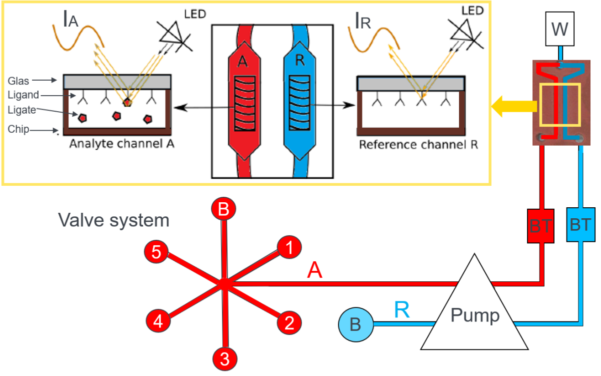

Fig. S2 Simplified representation of the microfluidic system. Liquid handling system consists of a peristaltic micro pump, which generates volume flow in reference line (R) and analyte line (A). The analyte line contains a 6-way valve to allow a sequential measurement of five samples plus buffer solution (B). Both lines are equipped with a bubble trap (BT) to filter out air bubbles of the fluidic system. The SCORE chip includes two micro channels with two entries for both lines and one common exit. Both channels are illuminated by a LED. The reflected light intensity I_R in the reference channel R is constant, whereas I_A in the analyte channel A changes due to the change in the optical thickness.

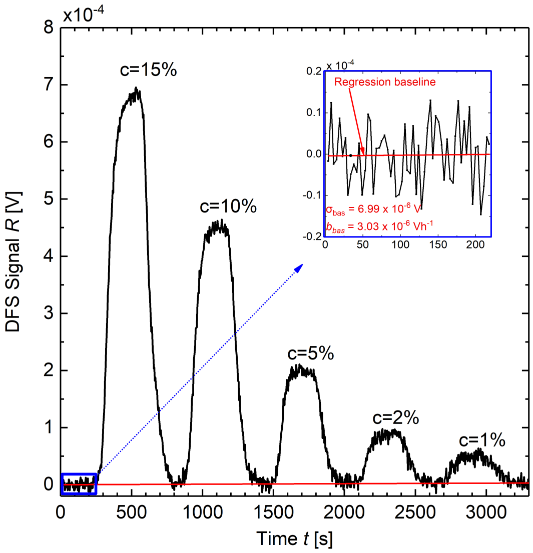

Fig. S3 Time resolved DFS Signal $R(t)$ of saline solutions with NaCl concentrations in ascending order c = 15%, 10%, 5% , 2% and 1%. For the cleaning of the system, distilled water is flushed between each saline solutions, e.g at 500 s, 1100 s and so on. The inset in the blue box shows the baseline analysis with small standard deviation of $\sigma_{\text{bas}} = 6.99 \times 10^{-6}$ V.

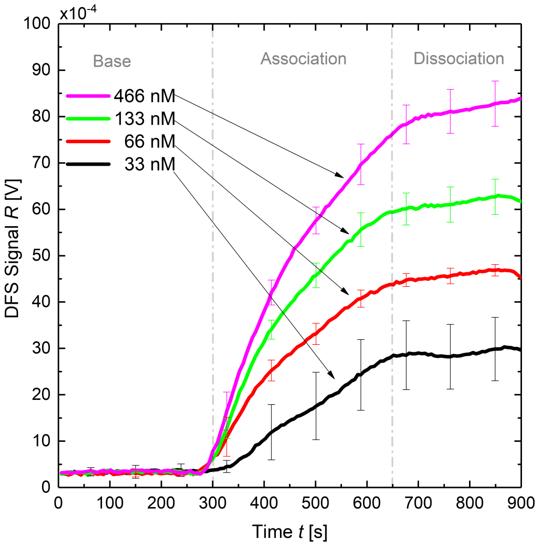

Fig. S4 Association and dissociation phases of antibodies solutions with concentrations $C_{\mathrm{A}} = 33$ nM, 66 nM, 133 nM and 466 nM. Error bars indicate the standard deviation of three-fold measurement for each concentration C_{A}. The beginning of the association phase starts at 300 s. After 650 s, buffer solution is applied to dissociate the antibodies. Due to the high affinity of the antibodies, a dissociation is not observable.

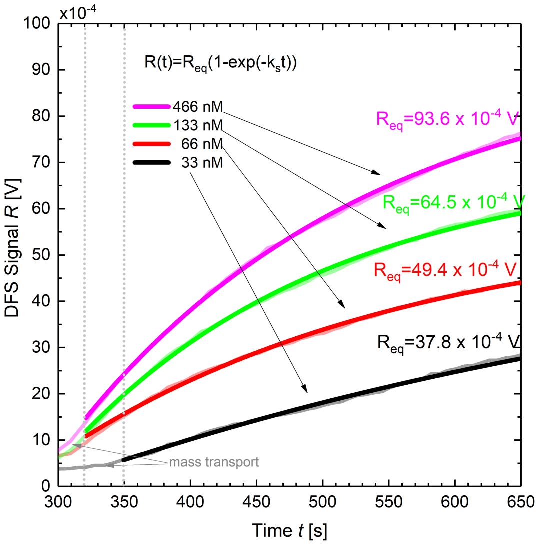

Fig. S5 Association phases of antibodies solutions with concentrations $C_{\mathrm{A}} = 33$ nM, 66 nM, 133 nM and 466 nM. The beginning of the association phase is controlled by mass transport. Therefore, we do not yet observe the predicted exponential saturation behavior. After 320 s for $C_{\mathrm{A}} = 66$ nM, 133 nM, 466 nM and 350 s for $C_{\mathrm{A}} = 33$ nM, the exponentially saturating kinetic part of the binding reaction can be identified as the rate determining step which can be fitted in order to determine R_{eq}.
